# Epitomics: Analysis of Plasma C9 Epitope Heterogeneity in the Plasma of Lung Cancer Patients and Control Subjects

**DOI:** 10.3390/ijms241814359

**Published:** 2023-09-21

**Authors:** Ilona Tornyi, Jozsef Lazar, Aladar Pettko-Szandtner, Eva Hunyadi-Gulyas, Laszlo Takacs

**Affiliations:** 1 Department of Human Genetics, Faculty of Medicine, University of Debrecen, 4032 Debrecen, Hungary; tornyi.ilona@med.unideb.hu; 2 Department of Pulmonology, Faculty of Medicine, University of Debrecen, 4032 Debrecen, Hungary; 3 Doctoral School of Molecular Cell and Immune Biology, University of Debrecen, 4032 Debrecen, Hungary; 4 Biosystems Immunolab Zrt., 4025 Debrecen, Hungary; jozsef.lazar@biosys-ilab.com; 5 Biosystems International Kft., 4025 Debrecen, Hungary; 6 Proteomics Laboratory, Biological Research Center, 6726 Szeged, Hungary; pettko-szandtner.aladar@brc.hu (A.P.-S.); gulyas.eva@brc.hu (E.H.-G.)

**Keywords:** epitomics, complement C9, epitope profiling, epitope heterogeneity, proteoform, biomarker, lung cancer

## Abstract

The human proteome is more complex than the genetic code predicts it to be. Epitomics, or protein epitome profiling, is a tool for understanding sub-protein level variation. With the ultimate goal to explore C9 proteoforms and their relevance to lung cancer, here we report plasma C9 epitope-associated molecular heterogeneity in plasma samples of lung cancer patients and control subjects. We show three C9 epitopes (BSI0449, BSI0581, BSI0639) with markedly different association with lung cancer (“unaltered”, “upregulated” and “downregulated”). In order to exclude confounding effects, we show first that the three epitope-defining mAbs recognize C9 in purified form and in the natural context, in the human plasma. Then, we present data demonstrating the lack of major epitope interdependence or overlap. The next experiments represent a quest toward the understanding of the molecular basis of apparent disparate association with lung cancer. Using immunochemistry, SDS PAGE and LC-MS/MS technologies, we demonstrate that epitope-specific immunoprecipitates of plasma C9 seem identical regarding peptide sequence. However, we found epitope-specific posttranslational modification and coprecipitated protein composition differences with respect to control and lung cancer plasma. Epitope profiling enabled the classification of hypothetical C9 proteoforms through differential association with lung cancer.

## 1. Introduction

**Figure 1:**
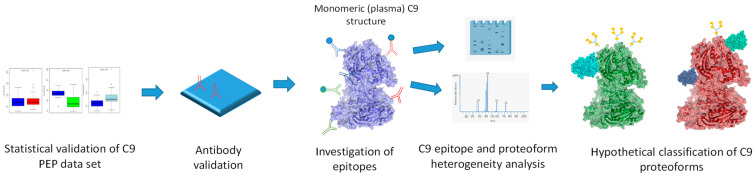
Workflow of this study showing the sequence of experiments. (Three-dimensional structure image of monomeric plasma C9 is from PDB ID C6Xo and it is based on a publication by Spicer et al. [9]).

The proteome is several orders of magnitude more complex than the coding capacity of the genome would predict [1,2]. Consequently, and as proteins are the main actors of biological function, understanding protein variability on the global scale is of key importance for gaining relevant biochemical information on protein variants and their activity [3]. Large-scale proteomics technologies are severely limited by approaches that look at proteins as single entities. Accordingly, when proteome variability is addressed by mass spectrometry and some of the affinity proteomics approaches on the global scale, the outcome of the measurements is not fully interpretable beyond protein units. Among others, this is well reflected by the approaches of the Protein Atlas [4,5] or the efforts of the Proteoform Consortium [6,7]. To explore epitome variability at sub-protein level, we recently reported that large-scale protein epitome profiling (PEP) [8], a technology that is hypothesis free and demonstrates previously undetectable biomarker granularity with regard to association with cancer, also delivers tools, epitope specific mAbs, to explore structural and functional relevance. In this report, we test epitope-specific mAbs of plasma C9 for which protein we previously detected epitope-dependent association with lung cancer (LC) [8]. Epitope variability may be due to structural variation due to alternative translational initiation and termination, alternative splicing, posttranslational modification, degradation, regulated processing and complex formation. Here, we present initial biochemical analysis of C9, displaying three different epitopes in the plasma of LC patients and control (Ctrl) subjects and ask the question, which, if any, of the potential epitope altering mechanisms are responsible for the variability. The data we present are then used to build hypothetical C9 proteoforms that show differential association with LC. The workflow of the experiments is shown in Figure 1.

## 2. Results

### 2.1. Statistical Validation of C9 PEP Data

**Figure 2:**
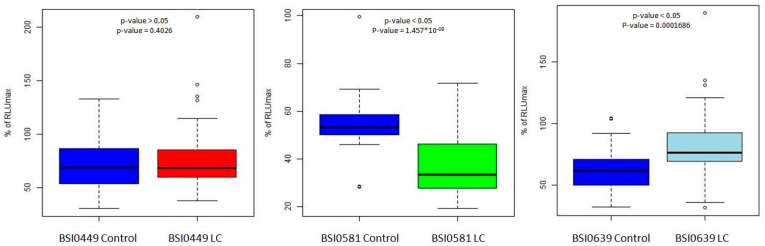
Three epitopes recognized by the tested mAbs were used for statistical analysis of C9 PEP data set [8] via Student’s *t*-test. The boxplots show relative representation of C9 epitopes (BSI0449, BSI0581, BSI0639) in individual samples of 425 LC patients and 433 matched controls. Technology: sbCIA on biochips (printed microarray), run on the Randox Evidence Investigator. Y axis shows % RULmax (100× RLU/RLUmax) values. X axis shows Ctrl and LC groups. (RLU: relative light unit).

In a recent publication [8], we reported the PEP dataset, including C9 epitomic LC association data, as receiver operator curves. In those experiments, LC association was tested in a single-binder capture inhibition assay (sbCIA) that runs on the Randox Evidence Investigation platform. In the sbCIA assay, epitope-specific BSI mAbs are immobilized on the three-dimensional porous ceramic matrix of the biochips. Next, the mix of the preincubated diluted plasma sample and the biotinylated tracer is contacted with the biochips, then, bound tracer is visualized via avidin HRP. From the previously published PEP data set, C9 epitomic data were statistically validated using Student’s *t*-test for mAbs BSI0449, BSI0581 and BSI0639. The results, including *p*-values, are shown as boxplots in Figure 2. The plasma representation of the epitope BSI449 shows no difference between the groups of LC patients and Ctrl subjects; therefore, hereinafter for simplicity, we designate BSI0449 epitope LC neutral. In contrast, BSI0639 shows significantly higher values in sbCIA; therefore, C9 protein carrying BSI0639 epitope is represented at a lower level in LC plasma samples, while BSI0581 presents an opposite pattern—its representation is higher in LC.

### 2.2. Validation of Monoclonal Antibodies

**Figure 3:**
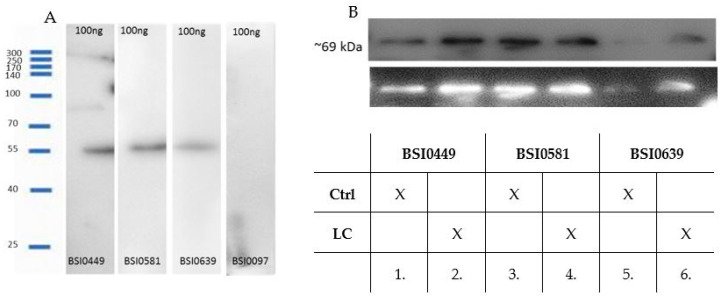
(**A**) Western blot with anti-C9 primary mAbs (BSI0449, BSI0581, BSI0639) and C9 protein (Sigma), anti-albumin mAb (BSI0097) was used as negative control. (**B**) Western blot of immunoprecipitates with anti-C9 mAbs (1:1 mixture of BSI0449:BSI0639) as primary mAb and GAM-HRP as secondary mAb. The reaction was developed by luminescence imager (B&W reversed image is shown on the lower panel). Lane 1: IP sample with BSI0449 mAb of Ctrl plasma. Lane 2: IP sample with BSI0449 mAb of LC plasma. Lane 3: IP sample with BSI0581 mAb of Ctrl plasma. Lane 4: IP sample with BSI0581 mAb of LC plasma. Lane 5: IP sample with BSI0639 mAb of Ctrl plasma. Lane 6: IP sample with BSI0639 mAb of LC plasma (Figure 3A,B full image is in Supplementary Figure S1A,B).

In order to demonstrate that BSI0449, BSI0581 and BSI0639 mAbs recognize C9, reducing SDS-PAGE followed by Western blotting was performed. Natural purified C9 (Sigma) was used as an antigen. The results are shown in Figure 3A and demonstrate that BSI0449, BSI0581 and BSI0639 recognize natural purified C9. It is important to note that at ~55 kDa the observed C9 band is a partially degraded fragment of C9 [10]. Next, immunoprecipitation via individual C9 epitopes followed by Western blot experiments were performed on pooled plasma samples, including 167 Ctrl subjects and 207 LC patients. Images shown in Figure 3B indicate that BSI0449, BSI0581 and BSI0639 recognize the natural full length C9 [11]. Although less intensive for BSI0639, each immunoprecipitate contains detectable C9 with similar molecular mass in both Ctrl and LC plasmas.

### 2.3. Investigation of C9 Epitopes

**Figure 4:**
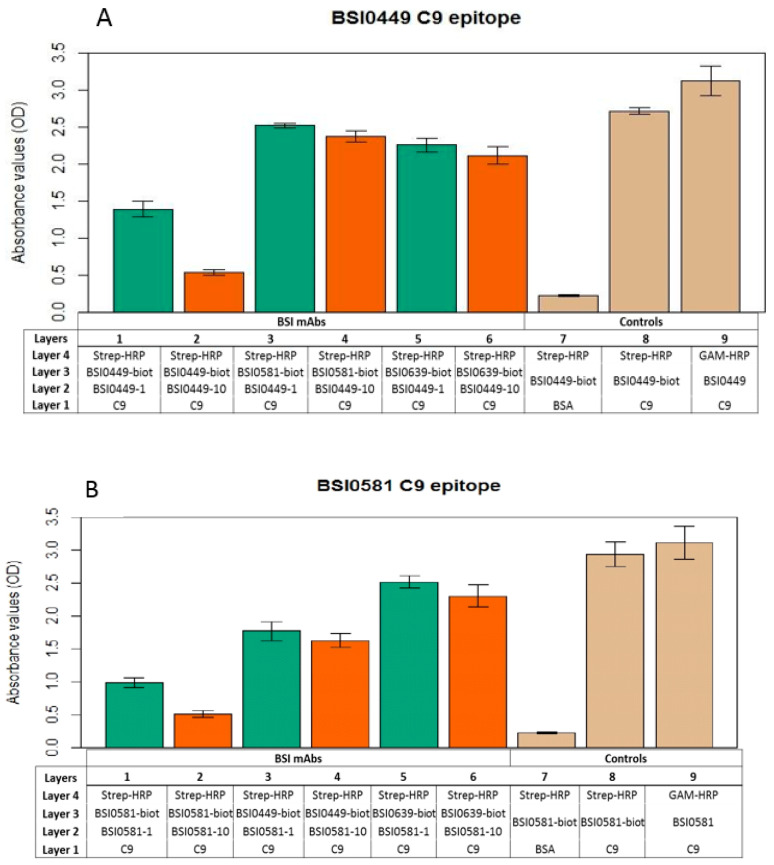

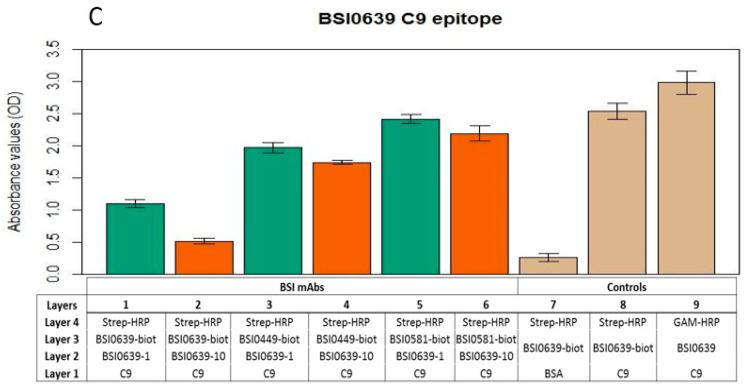
Result of BSI0449 (**A**), BSI0581 (**B**), and BSI0639 (**C**) anti-C9 mAb (at 1 µg/mL (green) and 10 µg/mL (orange) concentrations) epitope competition experiments and controls (tan). Table shows the different layers and incubation steps in the ELISA experiments. Each bar represents an average of four wells and SD is shown.

In order to explore whether the epitopes recognized by mAbs BSI0449, BSI0581 and BSI0639 are independent, mAb binding-competition ELISA experiments were performed. In these experiments, purified natural C9 (Sigma, St. Louis, MO, USA) was coated to wells of polypropylene plates and biotinylated BSI0449, BSI0581 and BSI0639 mAb recognized the antigen and provided strong signal following detection via streptavidin-HRP and chromogen in the presence of hydrogen peroxide as a substrate. Each mAbs (BSI0449, BSI0581 and BSI0639) was tested in this setup for inhibition at 1 µg/mL and 10 µg/mL concentrations against the biotinylated mAbs. As shown in Figure 4A–C, dose-dependent inhibition (>50%) is detectable exclusively with the corresponding non-biotinylated mAbs, the non-corresponding mAbs do not display strong inhibition (i.e., >50%). Small, but significant inhibition (<25%) with respect to the BSI449 epitope, by mAb BSI0581 was detectable. Non-specific binding was tested on bovine serum albumin coat; reactivity of both biotinylated and native mAb-s was independently tested on C9 coat (Figure 4A BSI0449, Figure 4B BSI0581, Figure 4C BSI0639). The epitope competition experiments indicate that the mAbs BSI0449, BSI0581 and BSI0639 react with three largely independent epitopes of C9. This is strongly supported also by available mimotope sequences published previously [8]. For clarity, the mimotope sequences are now shown in Supplementary Table S1. Minimal signal reduction, especially with BSI0581 in the context of BSI0449 binding, was observed (for *p*-values see Supplementary Table S2) and suggests that conformational changes induced by binding at one epitope (i.e., BSI0449) have some, albeit not strong, impact on binding to other epitopes (i.e., BSI0581).

### 2.4. C9 Epitope and Proteoform Heterogeneity in Control and Lung Cancer Subjects

**Figure 5:**
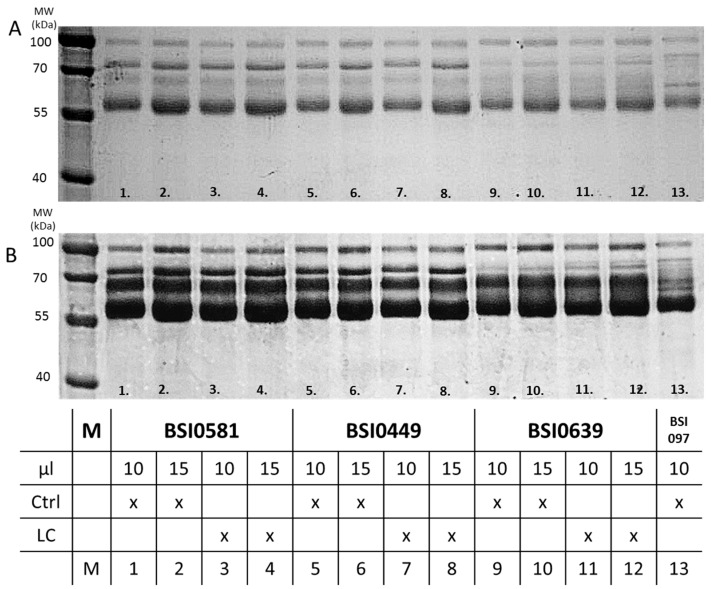
Immunoprecipitation of pooled EDTA plasma samples from Ctrl and LC subjects with anti-C9 mAbs (BSI0449, BSI0581, BSI0639). (**A**) Anti-C9 IP with Ctrl and LC plasma, SDS-PAGE, Coomassie G-250 staining. (**B**) Anti-C9 IP with Ctrl and LC plasma, SDS-PAGE, silver staining. Sample types are described in table below the figure (Full image is in Supplementary Figure S2).

To explore epitope-related molecular heterogeneity associated with biomarker performance of C9 displaying the different epitopes, first, we tested immunoprecipitates with each mAbs, BSI0449, BSI0581 and BSI0639 of Ctrl and LC plasma pools. Immunoprecipitates were loaded onto reducing SDS-PAGE gels in different volumes (10 µL and 15 µL). The gels were then stained with Coomassie G-250 blue first (Figure 5A), photographed, then de-stained and re-stained with silver [12] (Figure 5B). Immunoprecipitates of mAb BSI0639 were different from immunoprecipitates of BSI0449 and BSI0581, which did not differ from each other. In addition to the 69 kD band where C9 resides, BSI0639 precipitated relatively higher amounts of proteins with molecular mass 72 kD and <70 kD but >45 kD. There was no significantly visible difference when immunoprecipitates from Ctrl plasma were compared to that of LC plasma.

Because apparent differences were observed with respect to coprecipitated proteins, but the resolution of the reducing SDS-PAGE analysis was insufficient, immunoprecipitates from Ctrl and LC plasma samples with each of the mAbs (BSI0449, BSI0581 and BSI0639) were further analyzed by shotgun LC-MS/MS. Precipitates were digested in columns with trypsin. Aliquots of the tryptic digest were directly loaded for MS analysis (two independent experiments). Protein prospector analyses shows that each mAbs, BSI0449, BSI0581 and BSI0639 react with C9 because 3–13% of the precipitated tryptic peptides was of C9 with coverage from 30–45%.

**Figure 6:**
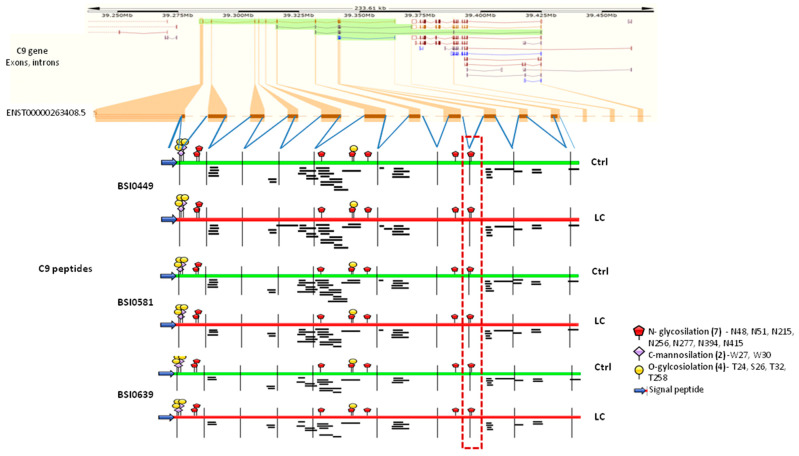
The graph shows C9 gene with its exon–intron organization (ENST00000263408.5). MS analysis-derived individual C9 peptides are shown under the exons (Ctrl samples, green; LC samples, red). Glycosylation modifications are based on the current literature [13,14] and UniProt database. MS C9 mapping analysis was performed by IBS illustrator online tools (version 1.0.) [15]. The dashed-line box outlines the N-glycosylated peptide (position 415), which showed marked difference in between the immunoprecipitates.

Differential epitope display could be the result of genetically coded structural variation, like alternative splicing, alternative translational start and termination. To assess the possibility that nucleotide sequence variance is responsible for the observed differential epitope display associated with biomarker quality, MS peptides from our dataset (https://doi.org/10.6084/m9.figshare.23659587) (accessed on 31 July 2023) were mapped to the C9 gene structure in the Ensembl database (ENST00000263408.5, https://www.ensembl.org/Homo_sapiens/Transcript/ProteinSummary?db=core;g=ENSG00000113600;r=5:39284140-39364495;t=ENST00000263408) for each of the mAbs (BSI0449, BSI0581 and BSI0639) and with respect to Ctrl and LC plasma samples. The comparison indicated that although the number of unique C9 peptides ranged from 27 (BSI0639 LC) to 40 (BSI0581 Ctrl), peptide mapping did not support the notion of genomically coded structural differences specifically associated with either one of the epitopes and/or the source of the plasma (Ctrl or LC) as shown on Figure 6. Comparison of the PeptideAtlas (https://peptideatlas.org/) (accessed on 3 November 2022) to our database of human C9 peptides indicates differences, namely the absence of signal peptides in our dataset, which is consistent with the plasma origin of C9 in our experiments. Moreover, due to technical differences, a large number of MS peptides are not detectable. Peptide counts of each MS detectable peptide were analyzed and displayed as aligned between Ctrl and LC (Supplementary Figure S3 and Supplementary Table S3). However, no notable differences were observed.

Epitopic variability may be due to posttranslational modifications. Therefore, we specifically searched for differentially represented N-glycosylated peptides in each sample set. We only found a single N-glycosylation difference at position 415. At 415 gly (HexNAc4Hex5NeuAc2; structure is shown in Supplementary Figure S4), the results indicate that in precipitates of BSI0499 (the LC neutral epitope), the ratio of 415 gly peptides between Ctrl and LC plasma is 1.25, while in the BSI0581 and BSI0639 precipitates, the ratio is 4.88 and 5.83, respectively (Table 1). On Figure 6, the dashed-line box shows the position of N-glycosylated peptide.

**Figure 7:**
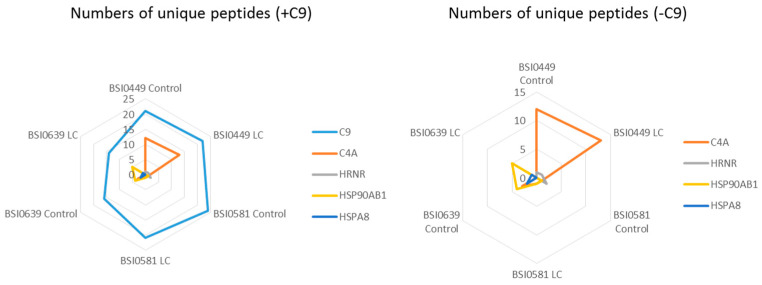
Radar plot shows the number of unique peptides of C9, C4A, HRNR, HSP90AB1, and HSPA8 in various immunoprecipitates (left panel, +C9). Right panel is a magnified view not showing C9 (−C9).

As in membrane rafts [16] and in solution as well, proteins circulate in complexes [17] kept together via weak interactions. Dynamic change in complex composition may be associated with biomarker relevance and function. Moreover, protein–protein association may mask epitopes by influencing availability to particular epitope-specific mAbs. To explore C9-associated proteins, a search was conducted for coprecipitated peptides. Figure 7 and Table 2 show unique peptide counts and coverage of coprecipitated proteins: C4A, HRNR, HSP90AB1 and HSPA8. Other proteins showed low level representation (<2 unique peptides) and were not included in the analysis. Surprisingly, BSI0499 mAb, which has neutral LC biomarker quality, coprecipitates 12 and 13 unique C4A peptides with 10.7% and 13% coverage from Ctrl and LC plasma, respectively. For BSI0581, the values are low, and there is no apparent difference between Ctrl and LC plasmas. In BSI0639, coprecipitates of C4A peptides were only found in the Ctrl plasma. The unique peptide and coverage values for HRNR are not remarkable; however, HSP90AB1 coprecipitated with BSI0639 mAb almost exclusively. The results are shown in Figure 7 as radar plots, and summarized in Table 2.

## 3. Discussion

Epitomics is a recently emerging omics technology that monitors dynamic changes of protein details, epitopes in health and disease. Using PEP technology, we reported the results of epitome profiling with mAb libraries as a rich source of cancer biomarkers [8].

Complement factor 9 is the major contributor to the terminal pore-forming complement complex that harbors C5b, C6, C7, C8. In the terminal complex, C9 units are polymerized. However, monomeric C9 units circulate and secure the necessary concentration of C9 molecules in the plasma and in tissues when the complement cascade is activated. Circulating monomeric C9 is prevented from polymerization by clusterin and vitronectin [18,19,20].

Plasma complement factor 9 has been identified as a biomarker for gastric, lung and colorectal cancers [21,22,23]; however, none of the previous reports dissected C9 to explore epitome-level association. We show that three independent epitopes of C9 recognized by mAbs (BSI0639, BSI0581 and BSI0449) exhibit markedly different associations with lung cancer: neutral (BSI0449), negative (BSI0639) and positive (BSI0581).

To better understand the underlying protein chemistry, here we report the analysis of immunoprecipitates of BSI0449, BSI0581 and BSI0639 mAbs from Ctrl and LC subjects.

Among others, epitopes may be influenced by genetically determined variation, posttranslational modification and complex formation. To explore these factors, first we analyzed the immunoprecipitates with SDS-PAGE and LC-MS/MS technologies.

SDS-PAGE analysis of the immunoprecipitates showed differences, especially with respect to BSI0639, and this directed our attention to protein complexes. To gain sufficient resolution, top-down, shotgun LC-MS/MS experiments were performed on the immunoprecipitates. Mapping MS peptides to the reported gene structure of C9 demonstrated that immunoprecipitates, regardless by which epitope and from which source were pulled down, showed identical distribution on the genomic map. Thus, C9 molecules were full length and most likely no structural difference could be identified or associated with either of the epitopes or sample source (Ctrl or LC plasma). The MS experiments strongly supported the Western blotting results from Ctrl and LC plasma, which showed similar molecular mass (69 kD), consistent with the molecular mass of C9. Moreover, MS peptides reported here covered 41.4% of the peptide sequence of soluble secreted C9. In contrast to the lack of detectable genomic sequence-based structural differences, we detected N-glycosylation variation in the samples.

N-glycosylation at position 415 showed [24] almost identical ratio between Ctrl and LC samples with respect to the “LC neutral” BSI449 epitope and mAb immunoprecipitates. Precipitation via the two non-neutral epitopes, BSI0639 and BSI0581, showed differential glycosylation (position 415 gly) ratio regarding LC status, 4.88 and 5.83, respectively. Thus, C9 molecules displaying epitopes BSI0639 and BSI0581 at position 415 were about five-fold more frequently glycosylated in Ctrl samples than in the LC samples.

Further heterogeneity was detected following the analysis of peptides coprecipitated with C9. Although we observed peptides representing a total of 78 proteins (Supplementary Table S4), here we discuss only those proteins with at least two unique peptides and detectable differences between Ctrl and LC. Complement factor C4A coprecipitated via the BSI0449 and BSI0581 epitopes, and HSP90AB1 protein coprecipitated via BSI0639 epitope are prominent because of the highest detected coverages, namely 13% and 9.8%, respectively. The LC-association neutral mAb, BSI0449, coprecipitated C4A at 10.3% coverage in Ctrl samples and 13% in the LC samples. While the non-neutral mAbs (BSI0639 and BSI0581) precipitates showed lower level or minimal coprecipitated C4A, BSI0639 coprecipitated C4A peptides from Ctrl plasma and none from LC plasma. BSI0581 C4 coprecipitates demonstrated negligible difference between Ctrl and LC samples. BSI0639 mAb co-precipitated HSP90AB1, and the peptides showed coverage of 9.8% and 7.7% coverage in the Ctrl and LC samples, respectively. BSI0581 precipitated less HSP90AB1 peptides, covering ~2% of the protein.

Taken together, epitome profiles are compatible with the existence of five hypothetical proteoforms shown in Table 3.

We suggest that proteoforms 2a and 2b are related and may represent variants produced by cells under stress or as a result of malignant transformation.

Further experiments are needed to explore whether the hypothetical proteoform classification is correct, and whether epitope expression is mutually exclusive or not. Whatever the case may be, C9 production in liver cells, monocytes and dendritic cells, thus in at least two different tissues, have been reported [25,26]. Complement 9 originating from different tissues may represent different proteoforms. Our search in the Proteome Atlas indicates C9 protein and mRNA expression in some cancer cells. Cancer-associated differential glycosylation has also been reported in multiple cancers [27] and specifically in lung cancer [28].

The results demonstrate that C9 epitope analysis via mAbs generates testable hypotheses regarding potential proteoform structures and their potential biomarker and/or functional capacity. Regarding the latter point, as a hypothesis, the following possibilities exist. Cancer tissue continuously activates complement and the terminal membrane attack complex is deposited onto the tumor’s outer surface, thereby killing cells which may release nucleic acid (DNA, RNA, etc.). The increased consumption of circulating C9 may, by a yet undefined mechanism, trigger C9 production in the liver and elsewhere. The “newly” synthesized C9 may display the molecular heterogeneity we observed, at least in part. Another possibility is that cancer cells produce C9, and this C9 is “different” from C9 produced by the liver. So far, we do not have data to support these suggestions.

## 4. Materials and Method

### 4.1. Pooled Plasma Samples

Control plasma pool (NCP) was prepared by mixing equal amounts from 167 individual plasma samples collected from healthy donors along with informed consents under the approval of the Regional and Institutional Ethics Committee of the Medical and Health Science Center, University of Debrecen (DE OEC RKEB/IKEB, permit no: 3049-2009, 3140-2010), Hungary. Individual EDTA plasma samples to the late-stage LC plasma pool (PCP), whose donors have late-stage (stage IV) lung cancer, were selected from a cohort assembled from four centers under the permit of the Medical Research Council of Hungary (ETT TUKEB, permit no: 11739/2014/EKU, 107/2014 and 417/2014). All participants had given their informed consent. PCP pool was prepared from 207 patients’ samples similarly to NCP.

### 4.2. C9 Immunoprecipitation

For immunoprecipitation, we used Dynabeads^TM^ Protein G (Invitrogen, Carlsbad, CA, USA). For each mAb conjugation reaction, 50 µL magnetic bead slurry was prewashed with 200 µL Wash and Binding (W&B) buffer (0.1 M Na_2_HPO_4_x12H_2_O (VWR, Lutherworth, Leicestershire, UK), 0.05% Tween (Sigma, St. Louis, MO, USA), pH 8.2). Then, 75 µg mAb dissolved in 200 µL W&B buffer was added to the prepared beads and incubated for one hour at room temperature (RT) with rotation at 5 RPM. Then, the beads were washed with 200 µL PBS buffer (123 mM NaCl (Spektrum 3D, Debrecen, Hungary), 3.16 mM KH_2_PO_4_ (Riedel-de Haën, Seelze, Germany), 10.45 mM Na_2_HPO_4_x2H_2_O (Riedel-de Haën, Seelze, Germany)), then with 200 µL 0.2 M triethanolamine (Sigma, St. Louis, MO, USA) in distilled water. After removal of the last washing solution, 200 µL of freshly prepared 20 mM DMP (dimethyl pimelimidate × 2HCl (DMP, Thermo Scientific, Waltham, MA, USA), in 0.2 M triethanolamine) was added to the beads. Beads were incubated for 30 min at RT with rotation at 5 RPM. The supernatants were removed and 200 µL of 0.2 M monoethanolamine (Sigma, St. Louis, MO, USA) in distilled water was added to incubate the mix for 15 min at RT with rotation at 5 RPM. Beads were washed three times with 200 µL PBS buffer after removing the supernatant.

Captured proteins were pulled down and isolated by mixing the beads with human plasma (200 µL pooled human plasma, 200 µL PBS buffer, 100 µL 20× protease inhibitor cocktail (1 tbl dissolved in 20 mL distilled water) (SIGMAFAST^TM^ Protease Inhibitor Tablets for General Use) (Sigma, St. Louis, MO, USA). Following overnight incubation and stirring at 4 °C, the beads were washed three times with 200 µL PBS buffer. Next, 100 µL PBS buffer was added to the beads. The bound material was eluted with 50 µL 50 mM glycine (Sigma, St. Louis, MO, USA) (diluted in distilled water, pH 2.5) after 10 min incubation at RT. The eluted sample was put into a clear Eppendorf tube which contained 10 µL 1 M Tris buffer (pH 9.0) (Sigma, St. Louis, MO, USA) solution.

### 4.3. SDS-PAGE

Reducing gels were prepared with ProSieve^TM^ 50 (Lonza, Basel, Switzerland) gel solution (12% resolving and 5% stacking) according to the manufacturer’s instructions.

The pulled down proteins were separated on 10 × 7 cm SDS-PAGE slab gel for one hour at 130 V.

### 4.4. Staining

SDS-PAGE gels were stained with Coomassie blue G-250 (Sigma, St. Louis, MO, USA), then with silver staining and visualized with GelLogic 2200Pro (Carestream, Rochester, NY, USA).

### 4.5. Western Blotting

SDS-PAGE electrophoresis was performed on C9 (Sigma, St. Louis, MO, USA, Product No. C3660). Then, proteins in SDS-PAGE gel were transferred onto polyvinylidene difluoride (PVDF) (Themo Scientific, Waltham, MA, USA) blotting membrane. WB was run in an ice-cooled transfer buffer (25 mM Tris, 192 mM glycine, 20% methanol) filled tank at 150 V for an hour. The membrane was then incubated in polyvinylpyrrolidone (PVP) (Sigma, St. Louis, MO, USA) blocking buffer (6.25 mM PVP, 18.8 mM NaCl in 50 mL PBS-Tween) overnight at 4 °C with shaking. On the following day, the membrane was incubated with anti-C9 primary mAb diluted in blocking buffer (10 ng/mL), overnight at 4 °C with shaking. Next, the membrane was washed 5 times with PBS-Tween. After washing, secondary antibodies were added, species-specific Goat anti-Mouse horseradish peroxidase (GAM-HRP (Invitrogen, Carlsbad, CA, USA)) diluted to 1:2500 in PBS buffer, then the membrane was incubated overnight at 4 °C with shaking. Next, the membrane was washed 5 times with PBS-Tween. The membrane was developed with Pierce ECL Western Blotting Substrate (Thermo Scientific, Waltham, MA, USA) according to the manufacturer’s guide and visualized with GelLogic 2200Pro (Carestream, Rochester, NY, USA).

### 4.6. Mass Spectrometry Analysis

#### 4.6.1. Sample Preparation

Total protein extracts were immunopurified using C9 epitope-specific antibodies (BSI0449, BSI0581, BSI0639) and protein G coupled magnetic beads with average particle size of 50 nm (MACS^®^ Technology, Miltenyi, Bergisch Gladbach, Germany) and digested in column with trypsin [29,30].

#### 4.6.2. Mass Spectrometry

An aliquot of the tryptic digest was analyzed by LC-MS/MS using a nanoflow RP-HPLC (Waters, Milford, MA, USA) (LC program: linear gradient of 3–40% B in 30 or 100 min, solvent A: 0.1% formic acid in water, solvent B: 0.1% formic acid in acetonitrile) on-line coupled to an Orbitrap-Fusion Lumos (Thermo Scientific, Waltham, MA, USA) mass spectrometer operating in positive ion mode. Data acquisition was carried out in data-dependent fashion, the 20 most abundant, multiply charged ions were selected from each MS survey for MS/MS analysis using HCD fragmentation (both spectra were acquired in the Orbitrap).

#### 4.6.3. Data Interpretation

Raw data were converted into peak lists using Proteome Discoverer (v 1.4) (Thermo Scientific, Waltham, MA, USA) and searched against the Swissprot database (downloaded 19 September 2017, 555,426 proteins) using Protein Prospector search engine (v5.15.1) (Thermo Scientific, Waltham, MA, USA) with the following parameters: enzyme: trypsin with maximum 2 missed cleavage sites; mass accuracies: 5 ppm for precursor ions and 10 ppm for fragment ions (both monoisotopic); fixed modification: carbamidomethylation of Cys residues; variable modifications: acetylation of protein N-termini; Met oxidation; cyclization of N-terminal Gln residues, phosphorylation of Ser/Thr/Tyr, allowing maximum 2 variable modifications per peptide. Acceptance criteria: minimum scores: 22 and 15; maximum E values: 0.01 and 0.05 for protein and peptide identifications, respectively.

Data were also searched for N-glycosylation: all common human N-glycan structures were allowed on N-glycosylation consensus motifs (NXT|S, X ≠ P).

Asn-415 glycosylation levels were assessed by comparing glycopeptide precursor ion intensities normalized to the most abundant unmodified C9 peptide LSPIYNLVPVK.

Spectral counting was used to estimate and compare relative abundance of individual proteins in samples [31].

### 4.7. mAb Biotinylation

A 10 mM solution of the biotin reagent, EZ-link Sulfo-NHS-LC-Biotin (Thermo Scientific, Waltham, MA, USA), diluted in double distillated water was prepared immediately before use. The biotin solution was added to the antibody solution (in a manufacturer’s instructions ratio). Then, the solution was incubated at room temperature for 30 min. For purifying the biotinylated mAbs we used Zeba^TM^ Spin Desalting Columns (Thermo Scientific, Waltham, MA, USA) (according to manufacturer’s guide, Zeba Desalting Handbook). To determine the biotinylated antibody quantity, we used Pierce Biotin Quantitation Kit (Thermo Scientific, Waltham, MA, USA), and to determine the concentration of biotinylated antibody we used Pierce BCA Protein Assay Kit (Thermo Scientific, Waltham, MA, USA).

### 4.8. C9 Epitope Test

The assay was performed on half-area ELISA plate (Costar3690, Corning, NY, USA), coated with 30 µL, 2 µg/mL of C9 (Sigma, St. Louis, MO, USA) mAb in carbonate coating buffer (15 mM Na_2_CO_3_ (Riedel-de Haën, Seelze, Germany), 35 mM NaHCO_3_ (Riedel-de Haën, Seelze, Germany), pH 9.6) for an hour at 37 °C. The plate was washed 2 times with PBS buffer, and blocked with 60 µL PBS-BSA-Tween buffer (NaCl (VWR, Lutherworth, Leicestershire, UK), BSA (Sigma, St. Louis, MO, USA), PBS buffer) for 30 min at 37 °C. The plate was washed three times with PBS buffer. Then, it was incubated with 30 µL non-biotinylated BSI anti-C9 mAb (BSI0449, BSI0581, BSI0639) in 1 and 10µg/mL dilution in blocking buffer for an hour at 37 °C. Then, the plate was washed 2 times with PBS buffer. Next, the plate was incubated with 30 µL, 1 µg/mL biotinylated BSI anti-C9 (BSI0449, BSI0581, BSI0639) mAb diluted in blocking buffer for an hour at 37 °C. After washing the plate three times with PBS buffer, it was incubated in 30 µL streptavidin labelled HRP (Southern-Biotech, Birmingham, AL, USA) diluted to 5000× in blocking buffer for 30 min at 37 °C. After washing the plate four times with PBS buffer, the reaction was developed with 30 µL TMB (Sigma, St. Louis, MO, USA) for 2 min. The reaction was stopped with 30 µL 4N H_2_SO_4_. The colorimetric changes were measured at 450 nm with FLUOstar OPTIMA plate reader (BMG LabTech, Durham, NC, USA).

### 4.9. Statistical Analysis

*p*-values were calculated by two-tailed *t*-test of significance (α = 0.05) after normality test (Shapiro–Wilk). The analysis was performed on R platform.

## Figures and Tables

**Table 1:** N-glycosylation ratio of protein C9 at 415 gly position and its ratio in Ctrl/LC pools.

Sample ID	Position 415 gly C9 Normalized Intensity to the Most Intense Unmodified C9 Peptide	Position 415 gly C9 Ctrl/LC Ratio
**BSI0449 Ctrl**	0.67	1.25
**BSI0449 LC**	0.54	
**BSI0581 Ctrl**	1.02	4.88
**BSI0581 LC**	0.21	
**BSI0639 Ctrl**	1.00	5.83
**BSI0639 LC**	0.17	

**Table 2:** Representation of proteins (with at least two unique peptides and differential representation between Ctrl and LC in at least one sample set) enriched in immunoprecipitates with different mAbs.

Samples ID	C9		C4A			HRNR			HSP90AB1			HSPA8		
	Num Unique	Peptide Count	Num Unique	Peptide Count	Coverage %	Num Unique	Peptide Count	Coverage %	Num Unique	Peptide Count	Coverage %	Num Unique	Peptide Count	Coverage %
**BSI0449 Ctrl**	21	312	12	48	10.7	1	2	0.8	0	0	0	0	0	0
**BSI0449 LC**	22	509	13	64	13	1	4	0.8	0	0	0	0	0	0
**BSI0581 Ctrl**	24	491	3	1	1.3	2	7	1.7	1	4	1.9	0	0	0
**BSI0581 LC**	21	332	3	1	1.3	0	0	0	1	4	1.9	0	0	0
**BSI0639 Ctrl**	16	139	6	3	4.4	1	1	0.8	4	14	7.7	2	7	3.4
**BSI0639 LC**	14	135	0	0	0	0	0	0	5	14	9.8	1	2	1.9

**Table 3:** Hypothetical C9 proteoforms.

	Epitope/mAb	C4A Association	C4A LC vs. Ctrl	HSP90AB1 Association	HSP90AB1 LC vs. Ctrl	415 gly	415 gly LC vs. Ctrl
**Proteoform 1**	BSI 449+	yes	no difference “high”	not detected	not relevant	no difference	LC neutral
**Proteoform 2a**	BSI 581+	yes	no difference “low”	no difference	no difference	different “high in Ctrl”	LC associated
**Proteoform 2b**	BSI 581+	yes	no difference “low”	no difference	no difference	different “low in LC”	LC associated
**Proteoform 3a**	BSI 639+	yes	no difference present in Ctrl only	no difference	no difference	different “high in Ctrl”	LC associated
**Proteoform 3b**	BSI 639+	no	not relevant	no difference	no difference	different “low in LC”	LC associated

## Data Availability

All MS raw data are available at figShare database (https://doi.org/10.6084/m9.figshare.23659587) (accessed on 31 July 2023).

## Supplementary Materials

The supporting information can be downloaded at: https://www.mdpi.com/article/10.3390/ijms241814359/s1.
