# Supplementary material for: Epitomics: Analysis of Plasma C9 Epitope Heterogeneity in the Plasma of Lung Cancer Patients and Control Subjects
Source: Int J Mol Sci. 2023 Sep 21;24(18):14359. doi: 10.3390/ijms241814359 (PMC10531758; doi:10.3390/ijms241814359)
Supplement: Supplementary file 1 [file ijms-24-14359-s001.zip › Tornyi et al._supplementary figures_v0920.pdf]

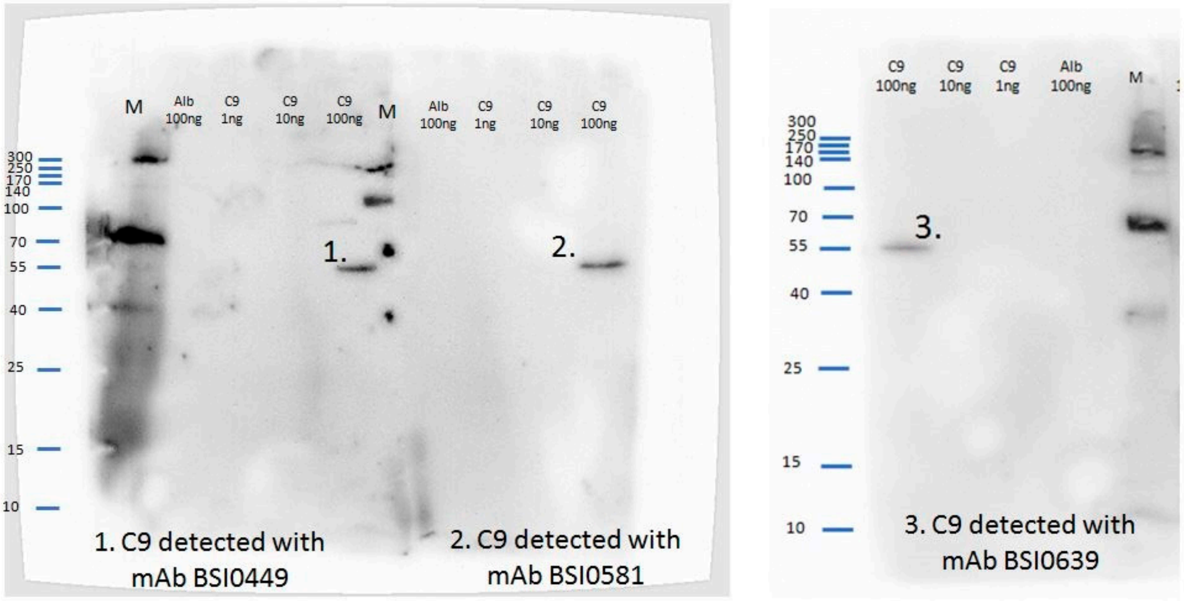

A)

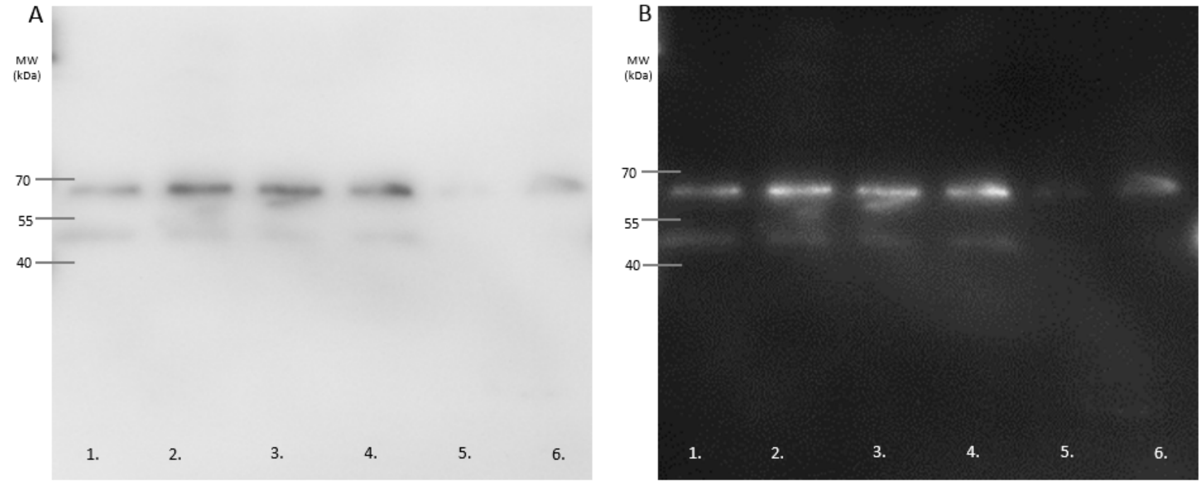

B)

Supplementary **Figure S1**: Full image of western blots. A) Upper figure panel: Western blot with anti-C9 primary mAbs (BSI0449, BSI0581, BSI0639) and C9 protein (Sigma), anti-albumin mAb (BSI0097) was used as negative control. (Full figure of Figure 3A.) B) Lower figure panel: Western blot of immunoprecipitates with anti-C9 mAbs (1:1 mixture of BSI0449:BSI0639) as primary mAb and GAM-HRP as secondary mAb. The reaction was developed by luminescent light (left) (developed by UV light is shown (right)) Lane 1: IP sample with BSI0449 mAb of Ctrl plasma. Lane 2: IP sample with BSI0449 mAb of LC plasma. Lane 3: IP sample with BSI0581 mAb of Ctrl plasma. Lane 4: IP sample with BSI0581 mAb of LC plasma. Lane 5: IP sample with BSI0639 mAb of Ctrl plasma. Lane 6: IP sample with BSI0639 mAb of LC plasma. (Full figure of Figure 3B.) Please note to presence of a specific faint band below 55 kDa mark, which is likely to represent the degraded and/or partially degraded fragment of C9 as we showed on Figure 3A.

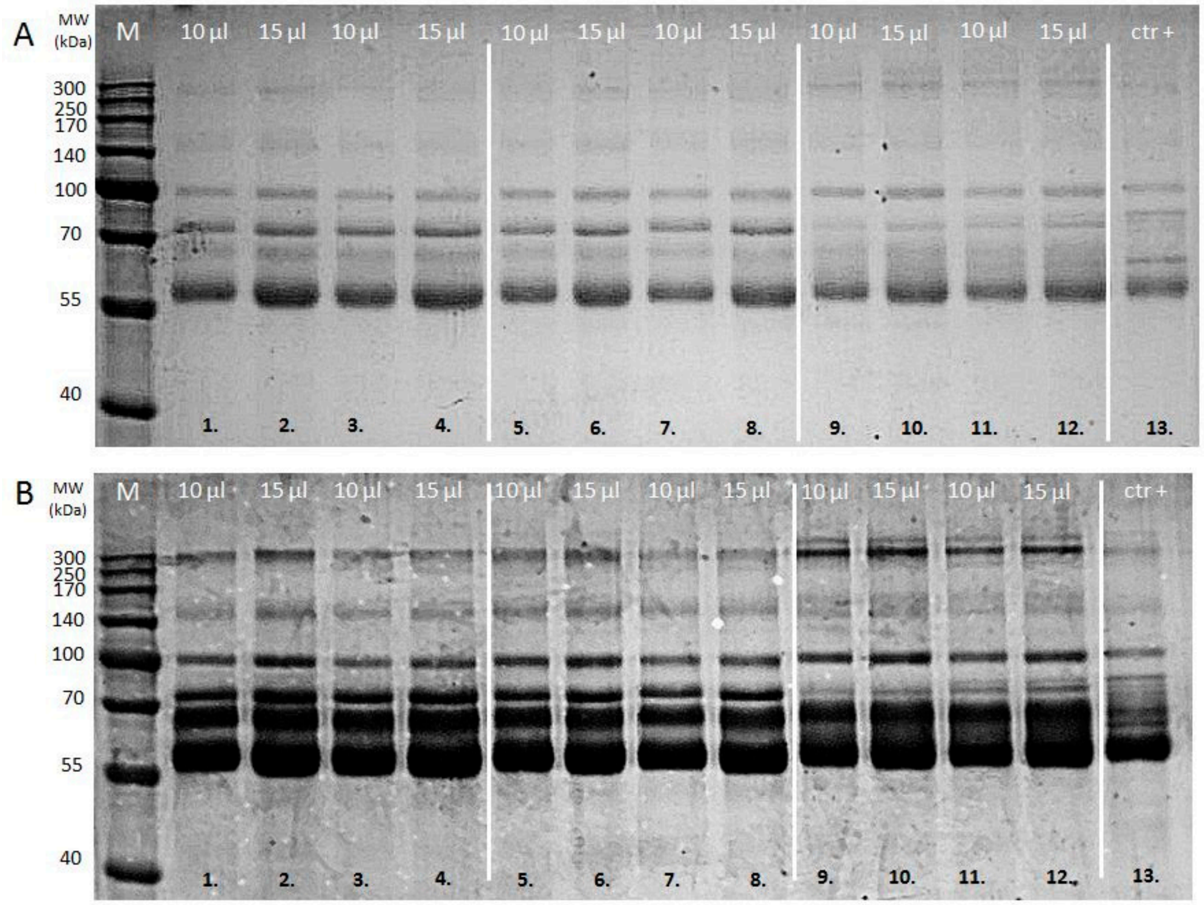

Supplementary **Figure S2:** Immunoprecipitation of pooled EDTA plasma samples from Ctrl and LC subjects with anti-C9 mAbs (BSI0449, BSI0581, BSI0639). (**A**) Anti-C9 IP with Ctrl and LC plasma, SDS-PAGE, Coomassie G-250 staining. (**B**) Anti-C9 IP with Ctrl and LC plasma, SDS-PAGE, silver staining. (Full figure of Figure 5.)

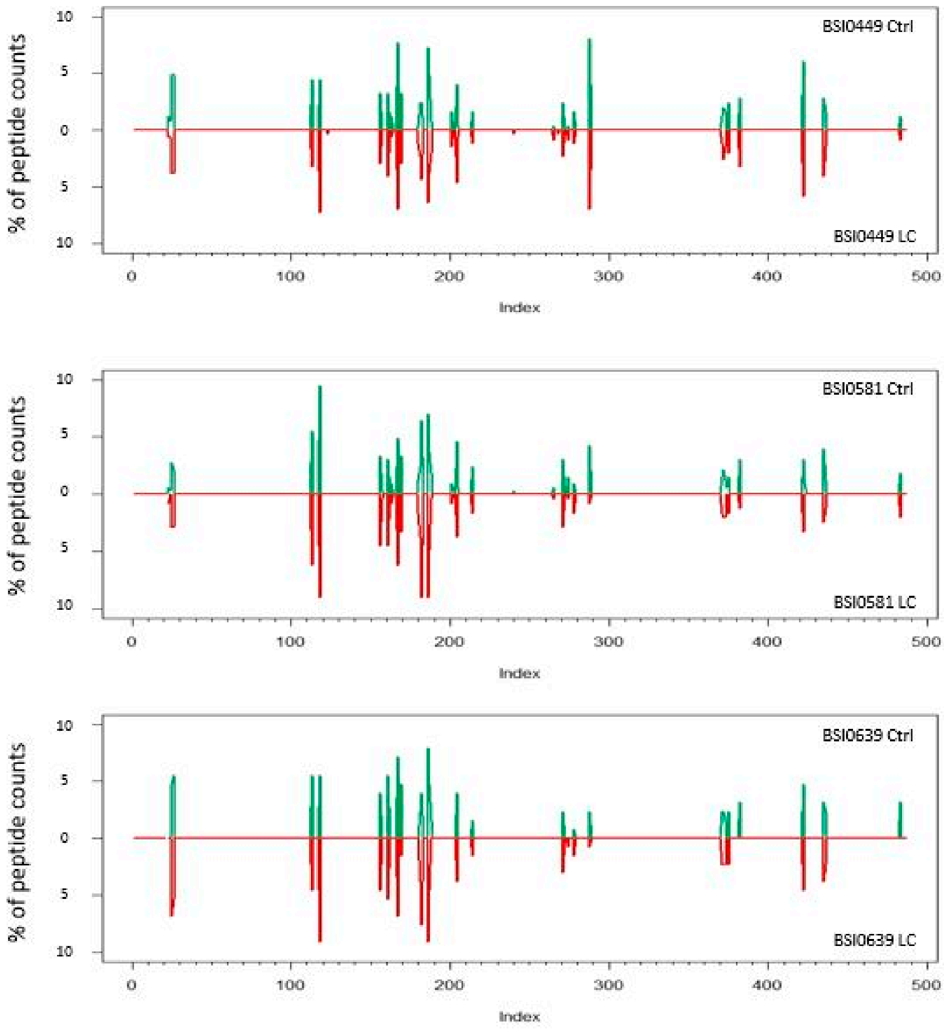

Supplementary **Figure S3**: relative peptide counts obtained from LC and control samples with three different immunoprecipitating antibodies. Observed peptide counts from our experiments are displayed in an ordered fashion from one to 487 (Supplementary Table2). Each position represents a C9 peptide in the Peptide Atlas database (https://peptideatlas.org/). Please note that positions where we did not observe any peptides, no vertical line is displayed. Lung cancer and control are virtually identical.

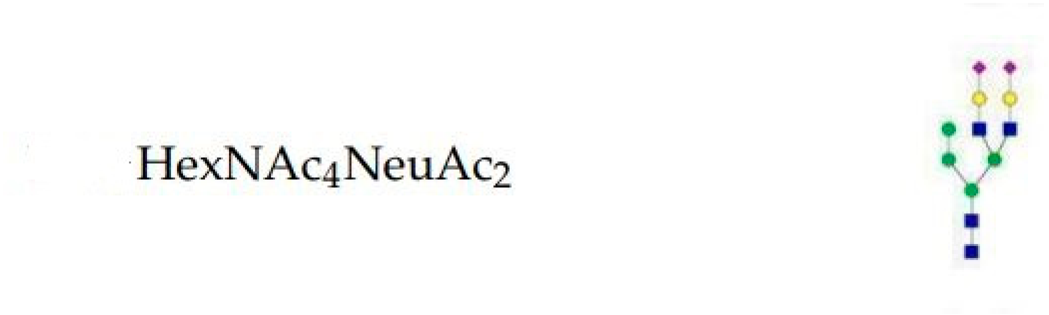

Supplementary **Figure S4**: Figure shows differentially represented N-glycan peptide 415 (HexNAc4Hex5NeuAc2).
